# Transient Force Measurement and Mechanism Analysis of Nanosecond Laser Ablation of Al/Ti Alloys Using Polyvinylidene Fluoride Sensors

**DOI:** 10.3390/s25092783

**Published:** 2025-04-28

**Authors:** Ming Wen, Baosheng Du, Luyun Jiang, Heyan Gao, Jianhui Han, Haichao Cui, Jifei Ye, Chenhui Yang

**Affiliations:** 1 State Key Laboratory of Advance Space Propulsion, Department of Aerospace Science and Technology, Space Engineering University, Beijing 101416, China; 2 Key Laboratory of Bio-Based Material Science & Technology, Ministry of Education, Northeast Forestry University, Harbin 100086, China

**Keywords:** transient force, nanosecond laser ablation, PVDF sensors, Al/Ti alloys

## Abstract

This study proposes a novel calibration method for polyvinylidene fluoride (PVDF) piezoelectric sensors based on electromagnetic force. The standard force source is obtained by calibrating the original force source of the inductor coil through an electronic balance. Transient force loading waveforms and peak values of PVDF piezoelectric sensors were obtained to analyze the mechanical effects of laser ablation on Al/Ti alloys. Transient force sensing using PVDF piezoelectric sensors exhibits a wide linear detection range (0.01–5.8 V) and high response values in response to changes in electrical signals. When irradiating Al/Ti alloy targets with different laser energies and spot sizes, the electrical signal intensity of PVDF piezoelectric sensors varies greatly, and the corresponding transient force peak value test results range from 0.01 to 8.5 N. This excellent transient mechanical sensing performance can be attributed to the high laser power density, efficient laser energy utilization, and the physical properties of the target material. COMSOL Multiphysics simulation results confirmed that the temperature and ablation center position of the surface of the target material undergo significant changes after being irradiated with different laser energies and spots. The simulation results are consistent with the experimental results. This research indicates that transient force measurements based on PVDF piezoelectric sensors have broad prospects in high-performance optical laser propulsion applications.

## 1. Introduction

Laser ablation includes the following stages: heating the target material, melting and gasification, plasma generation when the laser intensity is strong enough, and high-speed injection of high-temperature gas or plasma to generate thrust [1,2]. In laser ablation propulsion, gas [3], liquid [4], and solid [5] working mediums can all be used as ablated materials. The interaction between the laser and the target material and the conversion of laser energy into thruster power is a key process in achieving laser propulsion [1]. However, the thrust generated by the pulsed laser ablation of target materials is instantaneous and varies greatly with changes in laser parameters. Many working mediums have been used to study laser ablation propulsion, including pure water [6], metals [7], alloys [8], and energetic liquid working mediums [9]. Laser propulsion has received widespread attention due to its large thrust range, small unit impulse, excellent optical controllability, and excellent propulsion performance [10,11].

Polyvinylidene fluoride (PVDF) and its copolymers have excellent piezoelectric properties, good flexibility, easy processing, strong chemical stability, fast response time to input signals, a wide range of pressure measurements, adaptability to complex working conditions, easy embedding, and fast and simple operation [12,13,14]. Given the above advantages, PVDF has broad application prospects in the field of transient force measurement [15,16,17,18]. At present, the common calibration methods for PVDF piezoelectric sensors mainly include the Hopkinson bar method [19], the drop hammer calibration method [20], and the equal cantilever beam method [21]. However, there is still a lack of transient force calibration methods that are easy to operate and have a wide range of standard force source adjustments and high accuracy. In the measurement process for thrust generated through the laser ablation of a working medium, thrust measurement methods, such as the torsion pendulum method [22] and the force-induced luminescence method [23], cannot truly record the transient force waveform during pulse laser loading; thus, the measurement accuracy and measuring devices for these calibration methods need to be improved. It is of great significance to measure the transient force of laser ablation propulsion systems based on PVDF piezoelectric sensors and to analyze the laser ablation mechanism.

**Figure 1:**
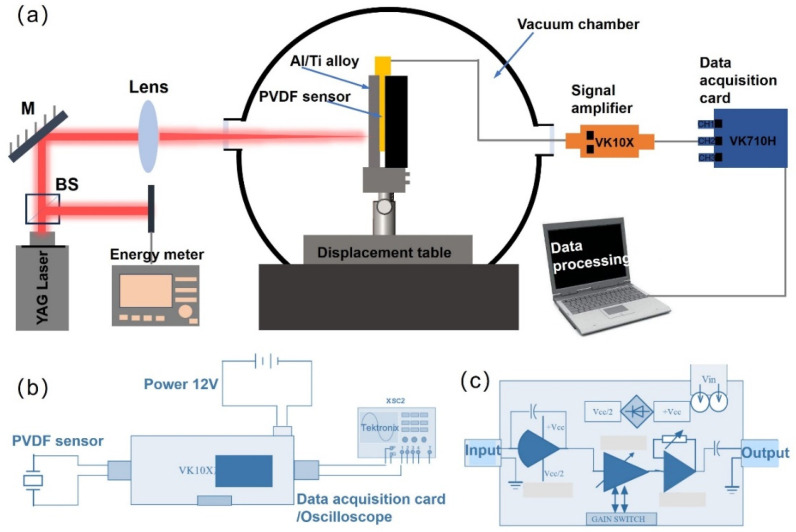
(**a**) Schematic diagram of a system for testing transient forces generated by laser ablation of Ti/Al alloy using PVDF piezoelectric sensors. (**b**) Circuit diagram of charge amplifier. (**c**) Circuit diagram of data acquisition card.

This study tests the transient forces generated by ablating Al/Ti alloy targets with different laser parameters using PVDF piezoelectric sensors under vacuum conditions. A transient force testing system (Figure 1a), a charge amplifier circuit (Figure 1b), and a data acquisition card circuit (Figure 1c) for the laser ablation of target materials are shown below. A novel calibration method for PVDF piezoelectric sensors based on electromagnetic force is proposed. The mechanical properties of Al/Ti alloy targets produced by laser ablation under different laser energies and laser spot size conditions were thoroughly studied, and the differences in mechanical effects produced by different laser ablation materials (Al and Ti alloy) were compared. The data indicate that the electrical signal intensity of PVDF piezoelectric sensors can be sensitively altered and exhibits high responsiveness and repeatability depending on laser parameters. The potential mechanism of thrust generated by the laser ablation of Al/Ti alloy targets under atmospheric and vacuum conditions is discussed in detail.

## 2. Materials and Methods

### 2.1. Calibrating Electromagnetic Force Source Using High-Precision Balance

As shown in Figure S1, we built an electromagnetic power source calibration platform, fixed the electromagnetic coil in the appropriate position inside of the balance (METTLER TOLEDO, Zurich, Switzerland) through an iron frame, and connected the electromagnetic coil power supply line. The electromagnetic coil was powered through a programmable power supply (IT6942A, ITECH ELECTRONIC Co., Ltd., Nanjing, China), which enabled us to change the electrical parameters of the coil by adjusting the power supply’s current. Subsequently, insulation pads were placed on the microbalance to prevent the electromagnetic force generated by the electromagnetic coil from affecting our ability to read the balance. A permanent magnet was placed on a cushion block, and the magnetic force generated by the electromagnetic coil was opposite to that of the permanent magnet, thereby generating a repulsive force. This repulsive force acts on a high-precision balance, changing the reading of the balance. Finally, we varied the electromagnetic force generated by the electromagnetic coil on the balance by varying the output current of the programmable power supply. We recorded the programmable currents and the corresponding balance readings and provided the magnitude of the electromagnetic force generated by a standard power supply under different electrical parameter conditions.

### 2.2. Testing the Coefficients of PVDF Piezoelectric Sensors Using Standard Force Sources

We built a calibration platform for the PVDF piezoelectric sensor and connected its signal acquisition circuit. Then, we placed the PVDF piezoelectric sensor on a carrier platform and a permanent magnet block above the sensor. We immediately used an iron frame to fix the electromagnetic coil, adjusted its position, and aligned it about 2 mm above the center of the permanent magnet. Finally, a programmable power supply was used to control the electromagnetic coil to output a fixed electromagnetic force. We applied a standard force source generated by the interaction of an electromagnetic coil and a permanent magnet applied to a PVDF piezoelectric transducer. Subsequently, we recorded the electrical signals of the transducer under different standard force source conditions. The piezoelectric coefficient of the sensor was obtained by linearly fitting a standard electromagnetic force to the peak voltage signal.

If the output voltage of the PVDF piezoelectric sensor measured by the calibration system is V(t), then its output current is

(1) $$i(t)=\frac{U(t)}{R}$$

Ignoring the capacitance of the circuit system (to the order of 10^−12^ F), the total output charge of the PVDF piezoelectric sensor is

(2) $$Q(t)=\int_{0}^{t}i(\tau )d\tau =\int_{0}^{t}\frac{U(t)}{R}dt$$

where *τ* represents the duration of the transient force generated by laser irradiation on an Al/Ti alloy on PVDF. Thus, the variation in transient forces on Al alloys over time can be measured. Using the calibration system to measure multiple sets of V(t) signals and standard force (*F*) signals for curve fitting, we can obtain the corresponding relationship:

(3) F(t)=KV(t)

where *K* is the dynamic piezoelectric coefficient to be calibrated and *F*(*t*) is the transient force signal measured by the PVDF piezoelectric film sensor. According to Equations (2) and (3), the transient force signal can be obtained by converting the output V(t) signal of the measured PVDF piezoelectric film sensor. This is a method that enables rapid measurement of transient forces.

### 2.3. Morphology Characterization and Transient Force Measurements

The morphology of the alloy target sample after laser ablation was characterized using an optical microscope. We measured electrical signals and transient forces using PVDF piezoelectric sensors equipped with charge amplifiers and data acquisition cards. The mechanism of thrust generated by the laser’s interaction with the Al/Ti alloys was based on the magnitude of the transient force and the morphology of the laser ablation. The transient force signal and the peak value generated by the Al/Ti alloy laser ablation were tested under different nanosecond laser optical parameters. We arranged PVDF piezoelectric sensors in the vacuum chamber and placed them on a 5 mm thick Al alloy plate and displacement table substrate. Then, the circuit of the PVDF piezoelectric sensor was connected through a vacuum flange, and the electrical signals of the sensor were recorded and analyzed using a charge amplifier, a data acquisition card, and an industrial computer. Eventually, we adjusted the energy parameters and spot parameters of the YAG laser to ablate the Al alloy substrate. The transient force signal of the laser-ablated Al/Ti alloy was collected in real time using the PVDF piezoelectric sensor. We then used the piezoelectric coefficient obtained in the above steps and the measured voltage peak value to test the transient force generated by this alloy.

### 2.4. Material Models and COMSOL Multiphysics Simulation

We chose nanosecond laser ablation of an Al/Ti alloy target as the research object and the surface of the target as the medium to study the mechanism of the pulse force generated by laser ablation. According to the laser irradiation characteristics, a two-dimensional finite element model was established using COMSOL Multiphysics 5.5. The calculated region was 4 mm in the *X* direction and 6 mm in the *Z* direction. The space occupied by the top was 1 mm, and the space occupied by the Al/Ti alloy target material was 4 × 5 mm. A pulse Gaussian laser source was applied to the top surface to simulate its propagation in the *X*-*Z* plane. The zero flux boundary condition was applied at the perimeter boundary. Convective heat flux boundary conditions were used on both sides. The initial conditions were an ambient temperature of 293.29 K and an ambient pressure of 1 bar. The laser parameters in the simulation were the same as those in the experiment. To simplify the calculation, we made the following assumptions: the flow of the liquid alloy target in the molten pool represents incompressible Newtonian laminar flow; the surrounding air is incompressible; the alloy target vapor is an ideal gas and transparent to the incident laser beam; the formation of plasma is not considered; multiple reflections are not considered; and the boiling point of the alloy target is independent of the pressure. Only heat transfer is considered during laser ablation.

When the nanosecond laser beam is irradiated on the surface of the Al/Ti alloy, the target and the surrounding environment are heated by heat conduction, convection, and radiation. With the increased temperature, under the action of laser energy absorption and recoil removal pressure, physical phenomena, such as vaporization, ejection, and boiling, occur in the melt, demonstrating the mechanism of the laser and material interaction. To explain the mechanism of pulsing force generated by the interaction between lasers and alloys, the general forms of the equations of the conservation of mass, momentum, and energy are used throughout the field of computation. The heat and fluid flow models are calculated as follows [24,25]:

(4) $$\nabla \vec{u}=0$$

where u is the molten velocity.

(5) $$\rho \left(\frac{\partial \vec{u}}{\partial t}+\vec{u}\cdot (\nabla \cdot \vec{u})\right)=\nabla \cdot \left[-PI+u(\nabla u+(\nabla \vec{u}))\right]+\rho \vec{g}+F+F_{\delta }$$

where *ρ* is the density of the material, *P* is the pressure, *I* is the identity matrix, *g* is the gravity acceleration, *F* is the body force, and $F_{\delta }$ is the surface tension force.

(6) $$\rho C_{p}(T)\left[\frac{\partial \vec{u}}{\partial t}+\nabla \cdot (\vec{u}T)\right]=\nabla \cdot (k(T)\nabla T)+Q_{L}$$

where *C_p_* is the specific heat capacity of the material; *T* is the absolute temperature; *k* is the thermal conductivity of the materials; and *Q*_L_ is the laser source term. The thermophysical properties of the target materials are shown in Table 1.

## 3. Results and Discussion

### 3.1. Electrical Signal Response and Coefficient of PVDF Piezoelectric Sensor

As shown in Figure S1, a calibration platform for an electromagnetic force standard source was built to simulate pulse force. The output current of the programmable power supply was adjusted to 0.01 A, 0.05 A, 0.5 A, 1.0 A, 1.5 A, 2.0 A, 4.0 A, and 8.0 A. A fixed electromagnetic force was applied to a balance, and the current was adjusted each time. Using the balance, the standard forces under corresponding electrical parameter conditions were calculated as 0.011 N, 0.056 N, 0.552 N, 1.075 N, 1.564 N, 1.984 N, 4.514 N, and 8.589 N, respectively. The relevant data are organized in Table S1.

**Figure 2:**
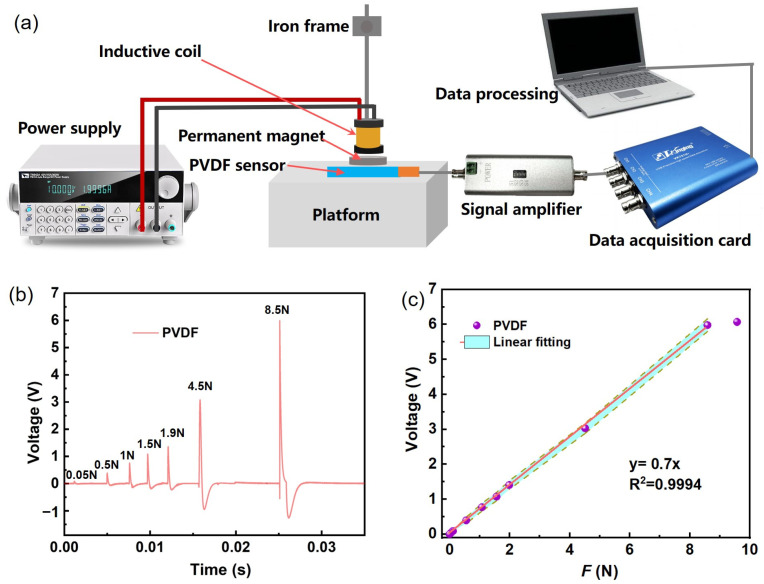
(**a**) System diagram of using the PVDF piezoelectric sensor to test standard force sources. (**b**) The relationship diagram of the voltage signal of the PVDF piezoelectric sensor over time. (**c**) Linear fitting graph between the magnitude of standard force and peak voltage.

To determine the mathematical relationship between voltage signals and transient forces, a PVDF piezoelectric sensor coefficient testing platform was built (Figure 2a). The output current of the programmable power supply was adjusted to a different value each time it was used. The waveforms of the piezoelectric signals were recorded by the sensor’s testing software (VK701H) under different electrical parameter conditions, and we readied the peak voltage of the piezoelectric signals. The piezoelectric coefficient of the sensor was obtained by linearly fitting the peak voltage under different standard force conditions, with the fitted line passing through the origin, and calculating the slope of the fitted line. Thus, the transient force magnitude of the laser ablation of different working mediums can be obtained by measuring the voltage signal of PVDF piezoelectric sensors. Thus, the PVDF piezoelectric sensors were successfully calibrated.

Figure 2a shows that the piezoelectric coefficient testing device of the PVDF piezoelectric sensor includes a programmable power supply (ITECH auto range to DC power supply; 60 V, 15 A, 360 W), a bracket for fixing the electromagnetic coil, an electromagnetic coil, a permanent magnet, an operating platform, a charge amplifier, a data acquisition card, and an industrial computer. Figure 2b shows the waveform of the voltage generated by the sensor under different electromagnetic force conditions as a function of time. The piezoelectric signal response of each standard electromagnetic force (0.05–8.5 N) condition is labeled in the figure. Figure 2c is a data diagram of the linear fitting between the peak voltage and the electromagnetic force. By adjusting the electrical parameters of the programmable power supply, a fixed standard force can be applied to the sensor. The electrical signal on the sensor is recorded on the testing software of the industrial computer through the charge amplifier and the data acquisition card. As shown in Figure 2b, when the output current of the programmable power supply is greater than 0.05 A, the piezoelectric sensor has a clear response signal, corresponding to a standard force of 0.056 N. When the output current of the programmable power supply is greater than 0.01 A, the piezoelectric sensor shows a recognizable voltage response signal (greater than twice the background noise), corresponding to a standard force of 0.011 N (Figure S2). When the electromagnetic standard force is less than 0.01 N, the piezoelectric signal value mixes with the back-bottom noise, and no significant response value appears, so the detection lower limit is set to 0.01 N. This lower detection limit is mainly attributable to the control of ambient noise in the vacuum chamber and the effective utilization of the stabilized platform. As shown in Figure 2c, the peak voltage obtained by loading the corresponding standard force and electromagnetic standard force onto the PVDF piezoelectric sensor is linearly fitted, and the fitted line passes through the origin. The relationship between the peak voltage of the sensor and the standard force source is y = 0.7x, and the piezoelectric coefficient of the sensor is 0.7. The R^2^ is 0.9994 and the area of the green dashed line is the error bar chart. Therefore, the transient force value generated via the laser ablation of a working medium can be calculated based on the peak voltage of the PVDF piezoelectric sensor being tested. When the electromagnetic force is greater than 8.5 N, the increased piezoelectric signal change is not obvious and does not increase linearly with an increase in electromagnetic force. When the electromagnetic force is set to 9.5 N, the peak voltage of the piezoelectric signal is about 6 V, which is slightly higher than the value (5.9 V) of the voltage signal response when the electromagnetic force is 8.5 N (Figure 2c). This phenomenon mainly stems from the saturation characteristics of PVDF piezoelectric sensors. In practice, the linear detection range is the most important evaluation criterion for sensors. This sensor has a linear detection range of 0.01–8.5 N.

### 3.2. Spot Morphology of Laser Ablation of Al/Ti Alloy Samples

**Figure 3:**
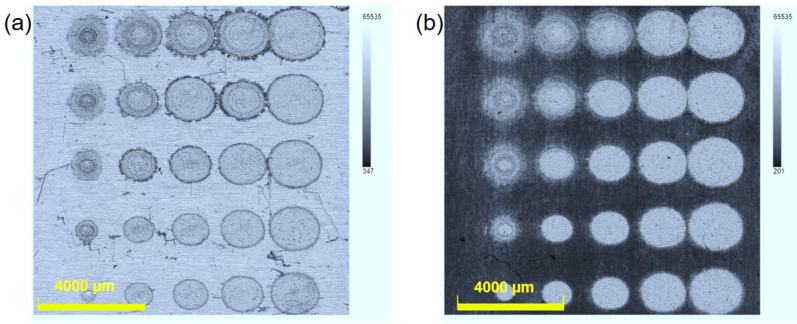
Microscopic morphology of nanosecond laser ablation of Al/Ti alloy samples with different laser parameters: (**a**) Al alloy sample; (**b**)Ti alloy sample.

The morphologies of the Al/Ti alloy samples ablated using laser parameters with different energies and focus spot sizes were characterized using a 3D depth of field microscope. Figure 3a,b show a top view of the microscopic images of the laser-ablated alloy targets with laser energies of 800 mJ, 500 mJ, 350 mJ, 150 mJ, and 90 mJ; the laser-ablated Al/Ti alloy target samples exhibit obvious elliptical pits, and the ablation spot size is calculated according to the short axis direction of the ellipse, with an average laser spot size of ~1.48 mm, ~1.24 mm, ~1.02 mm, ~0.96 mm, and ~0.76 mm. The pit depth of the laser spot size varies with different laser parameters. When the laser spot is ~0.76 mm, it has the deepest pit and presents a clear hemispherical shape; conversely, when the spot size is 1.48 mm, the ablation pit is very shallow, mainly due to surface ablation. In contrast, the depth of the pits varies under different laser ablation conditions, while the spot morphology of the Al and Ti alloys ablated with the same laser parameters remains basically the same.

### 3.3. Electrical Signal Response of Al/Ti Alloy Ablated Using Different Laser Parameters

**Figure 4:**
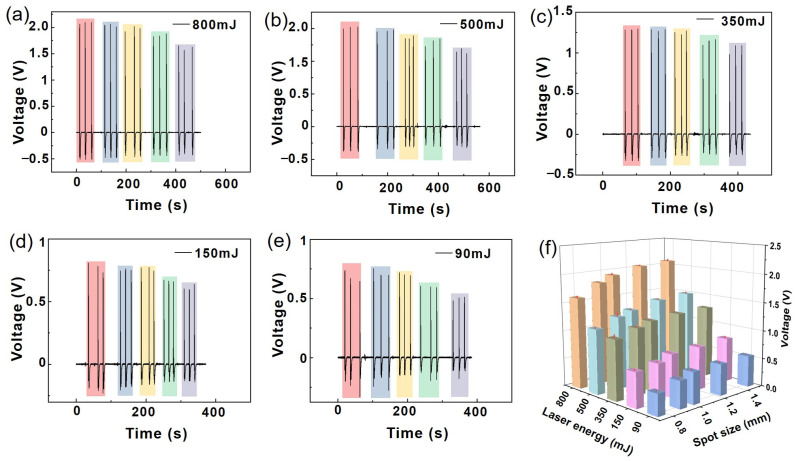
Voltage signal generated by laser ablation Al alloy of PVDF piezoelectric sensor under different spot sizes with laser energy of 800 mJ (**a**), 500 mJ (**b**), 350 mJ (**c**),150 mJ (**d**), and 90 mJ (**e**). Diagram of peak voltage variation with laser energy and spot size (**f**). ((**a**–**e**): Red, blue, yellow, green, and gray represent spot sizes of ~1.48 mm, ~1.24 mm, ~1.02, ~0.96 mm, and ~0.76 mm, respectively).

To study the transient force characteristics of using laser ablation on alloy targets, PVDF piezoelectric sensors were used to analyze the interaction between different laser parameters and alloy targets. Under vacuum conditions, a single-nanosecond pulse laser was used to ablate Al alloy substrate targets in a vacuum chamber, and voltage signal changes were used to control the output energy and spot size of a YAG laser. The size of the laser ablation spot on the surface of the Al/Ti alloy plate was adjusted using an optical focusing system. The sensors recorded the voltage signals generated by the ablating Al/Ti alloys, and the peak values of the voltage signals were recorded. The magnitude of the transient force was calculated based on the piezoelectric coefficient of the PVDF sensor. The transient force change process generated by Al/Ti alloy laser ablation was evaluated based on the sensor’s voltage waveform signal. Figure 4a–e summarize and analyze the voltage signals of the PVDF piezoelectric sensors obtained under different laser energy and laser spot size parameters. The experimental test of the laser ablation of the Al alloy working mediums was repeated three times for each laser energy and corresponding spot parameters. The peak voltage signal values under the same laser ablation conditions were slightly different, which was mainly caused by error sources, such as the target surface, sensor noise, and environmental fluctuations. The voltage peak values under different laser energy and spot size optical parameters are summarized in Figure 4f. The experimental results show that the peak voltage signal increased from 0.56 V to 2.08 V with the increased laser spot size and energy, with corresponding peak transient force values in a range of 0.8–3 N. It can be inferred that the transient force generated by the laser ablation of an Al alloy working medium also increases with an increase in laser energy and spot size. This is because the high-power-density laser (>10^9^ W/cm^2^) creates effective body ablation in the Al alloy, which generates significant thrust. There was no significant signal response from the PVDF piezoelectric sensor when the laser energy was further reduced (<90 mJ). This can mainly be attributed to the fact that most of the laser energy was consumed by the reflection from the metal. Therefore, Al alloy laser ablation produces significant thrust when sufficiently high laser power density is required.

To study the transient force generated by the laser ablation of different target materials, we chose Ti alloy for comparative research owing to its high strength, low density, high temperature, corrosion resistance, and other excellent characteristics, as well as its wide applications in spacecraft system components. The electrical signals of the PVDF piezoelectric sensors obtained using Ti alloy laser ablation (using the same parameters as with Al alloys) are shown in Figure S3a–e. The peak voltage values under various laser ablation conditions are summarized in Figure S3f. The experimental results show that as the laser energy decreases, the electrical signal intensity of the sensors also weakens, indicating that the stronger the laser energy, the better the thrust effect. Conversely, under the same laser energy conditions, as the laser spot size changes from large to small, the piezoelectric signal intensity first increases and then decreases, mainly due to the different laser power densities caused by the different spot sizes. On one hand, when the laser spot is large, the laser power density is small, making it difficult to effectively ablate the alloy target material; thus, the migration mass caused by the surface ablation of the target material is also small. On the other hand, when the laser spot is small, the high intensity of the Ti alloy results in less migration mass. However, when the laser energy is high enough, the laser power density is higher, and the ablation effect on the Ti alloy target is superior. Therefore, when the laser energy is 800 mJ or 500 mJ, the electrical signal intensity generated by Ti alloy laser ablation increases with a decrease in laser spot size. Thus, an optimal laser ablation condition occurs at a laser spot size of about ~1 mm with a different laser energy value.

**Figure 5:**
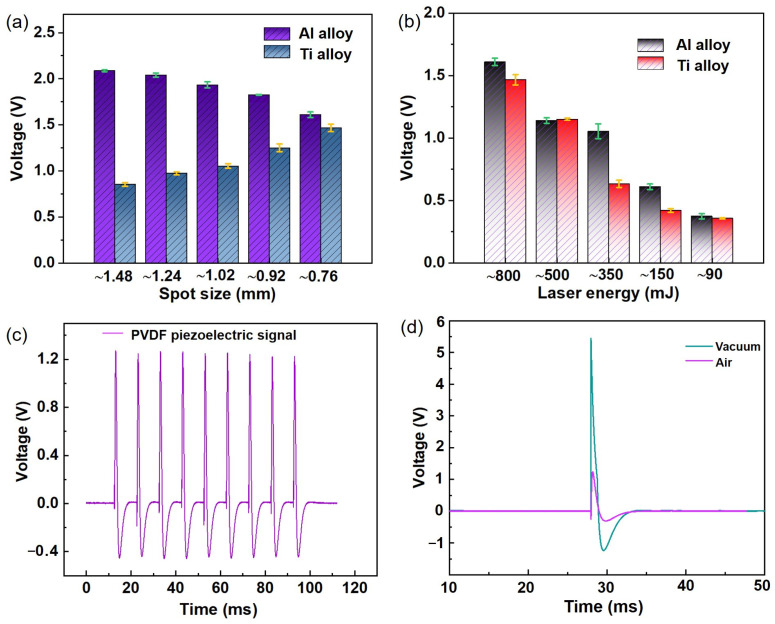
(**a**) Comparison of voltage signal output by PVDF sensors during laser ablation of Ti alloy under the same laser energy (800 mJ) and different spot sizes. (**b**) Comparison of voltage signal output by PVDF sensors during laser ablation of Ti alloy under different laser energies and the same spot sizes (0.76 mm). (**c**) Repeatability of voltage signal output by PVDF piezoelectric sensor under the same laser spot size and laser energy conditions. (**d**) Comparison of voltage signal output by PVDF piezoelectric sensors under vacuum and air conditions.

To explore the influence of laser ablation with different material targets on the generated transient force, the peak voltage signal intensities obtained by the PVDF piezoelectric sensor during Al/Ti alloy laser ablation under conditions using the same laser energy but different spot sizes and the same laser spot size but different laser energies are summarized in Figure 5a,b. The experimental results show that the peak piezoelectric signal intensity during Al alloy laser ablation decreases with a decrease in spot size; with Ti alloy, the signal intensity increases with an increase in laser spot size. Thus, the signal intensity of Al alloy laser ablation is obviously stronger than that of the Ti alloy. Notably, under different laser energy conditions, the peak piezoelectric signal intensity of Al and Ti alloy laser ablation decreases with a decrease in laser energy. These experimental rules are mainly due to the different basic characteristics of the different targets. The melting point of 6061 Al alloy is about 700 degrees, and its hardness is about 95–105 HV, while the melting point of Ti alloy is about 1660 degrees, and its hardness can reach 350–380 HV [26,27,28,29]. Targets with high melting points and strengths require very high laser power density for effective ablation, thereby increasing the migration quality of ablation formation and the piezoelectric signal strength of the transient force [8,30,31]. Therefore, Ti alloys require a higher laser power density. When the laser spot is large, it is not conducive to the formation of high laser power density, causing the piezoelectric signal intensity to increase with a decrease in spot size. However, when the spot size is small, a laser with high enough energy can produce high enough power density to generate large mass migration; thus, the peak piezoelectric signal intensity of Al/Ti alloy laser ablation will decrease with a decrease in laser energy.

During solid-target laser ablation, the mass ablation rate, laser absorption efficiency, and pressure characteristics of the vapor generated are the main factors affecting the magnitude of the transient force. Under high laser energy density, the plasma generated will affect laser absorption, and there will be no significant difference in laser absorption efficiency and vapor pressure. The ablation volume is estimated based on the volume of the laser ablation pit, and the migration mass of the laser-ablated alloy target is obtained. When the laser energy is 800 mJ, the ablation masses of Al alloy under different spot sizes are 392.46 μg, 315.33 μg, 220.19 μg, 180.5 μg, and 164.58 μg. The ablation masses of Ti alloy under different spot sizes are 209.37 μg, 174.19 μg, 127.45 μg, 119.86 μg, and 104.29 μg. The migration mass of Al alloy ablation is 1.5 to 1.9 times that of Ti alloy. The results show that the migration mass of the laser-ablated target is the most critical factor affecting the magnitude of the transient force. Therefore, under the same laser parameters in this experiment, the transient force generated by the Al alloy was significantly greater than that of the Ti alloy.

Figure 5c shows a repeatability test diagram of the voltage signal output by a piezoelectric sensor under the same laser spot size and energy values. The voltage signal curve of the sensor was obtained when the same laser parameters (350 mJ; 0.76 mm) were used to ablate the Al alloy. The figure shows that after nine measurements, the voltage signal of the sensor had good repeatability under the same conditions; the maximum peak value of the voltage was about 1.23 V, and the minimum peak value was 1.21 V. The deviation of the measured voltage was less than 1.7%. Figure 5d compares the voltage signal output by the piezoelectric sensor under vacuum and air conditions with the same laser parameters (350 mJ; 0.76 mm) to ablate the Al alloy. Compared with the air conditions, the peak voltage signal generated through laser ablation of an Al alloy working medium is much larger than under vacuum conditions, mainly because a certain amount of mass migration and a shockwave occur when the Al alloy target is irradiated with high laser power density. These factors generate significant momentum. Therefore, the jet resistance generated by the laser ablation of an alloy working medium under air conditions is relatively large, and the PVDF piezoelectric sensor is subjected to a greater reaction force.

**Figure 6:**
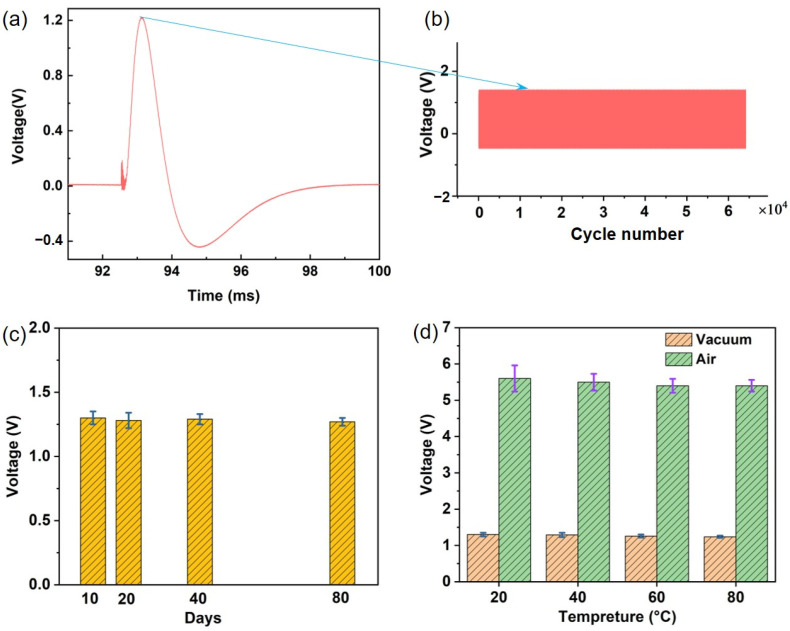
(**a**) Voltage signal output by PVDF sensors with single pulse laser ablation of Ti alloy. (**b**) Voltage signal output by PVDF sensors with 65,000 cycle numbers of laser ablation of Ti alloy. (**c**) The long-term durability and stability of voltage signal output by the PVDF piezoelectric sensor under the same laser spot size and laser energy conditions. (**d**) Comparison of voltage signal output by PVDF piezoelectric sensors with different vacuum and air conditions.

The long-term durability and stability of a PVDF sensor under repeated laser-induced shock loading are shown in Figure 6a,b, where the same laser parameters are used to ablate the Ti alloy. The piezoelectric signal changes in a single laser pulse ablating the Ti alloy target are shown in Figure 6a, which first presents a vibration signal caused by a photo-explosive explosion on the surface of the laser-ablated target. Subsequently, the products of the laser ablation target expand outward to perform their work and produce an obvious signal of transient force change with time. The piezoelectric signal values after 65,000 cycles are shown in Figure 6b. The results show that there is no obvious change in the signal after many cycles. The peak voltage signal changes within 0.6%, and the transient force sensor presents good long-term durability and stability.

Figure 6c,d show the time stability of an FVDF piezoelectric sensor and the effect of temperature on it, respectively. The experimental data show that 10, 20, 40, and 80 days after testing the same piezoelectric signal parameters, the response value of the variance is only 2.6%, indicating that the sensor under these working conditions has good time stability. The experimental results in Figure 6d show that the signal of the piezoelectric sensor decreases by only 3.5% with an increase in temperature in the air environment, and the piezoelectric signal decreases only 5.1% under vacuum conditions. These experimental results indicate that the piezoelectric sensor is insensitive to temperature change and has excellent temperature stability.

### 3.4. Transient Force Generation Mechanism of Al/Ti Alloy Laser Ablation

**Figure 7:**
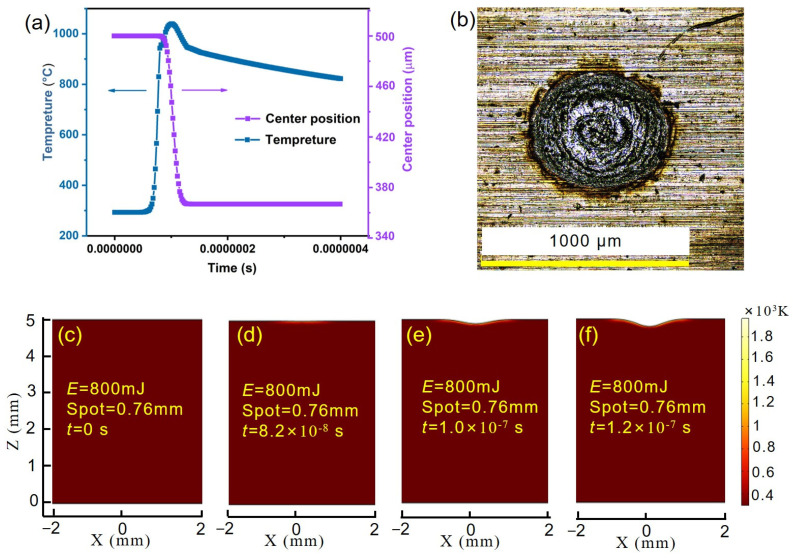
(**a**) Simulation of the temperature and center position variation relationship over time of laser ablation of Al alloys. (**b**) Microscopic morphology of nanosecond laser ablation of Ti alloy under laser energy of 800 mJ and spot size of ~0.76 mm. (**c**–**f**) Simulation of the process of center position and temperature change in laser ablation of Al alloy with laser energy of 800 mJ and spot size of ~0.76 mm.

To further analyze the influencing factors and mechanisms of Al/Ti alloy laser ablation used to produce transient force, the physical process of the target was simulated with the same parameters as the experiment. As shown in Figure S4, the single pulse laser waveform incident on the surface of the target is a nanosecond Gaussian beam. Figure 7a shows the relationship between the temperature of the laser ablation Al alloy target and the position of the spot center with time. The temperature increases significantly with the time of laser loading and then decreases slowly. The spot center position begins to decline rapidly and then stabilizes as the laser ablation threshold is reached. The whole process tends to stabilize in about 120 ns, and then the temperature of the laser ablative target spreads to the inside of the plate. When the melting point temperature of the Al alloy is reached, effective ablation will form. Over time, more of the material melts, causing the depth of the laser ablation pit to increase. Figure 7b shows a 2D microscopic image with a laser energy of 800 mJ and a spot size of 0.76 mm during the experiment. There are obvious hemispherical pits in the image, and the experimental results are similar to the simulation results. Figure 7c–f show the surface temperature of the laser ablation target and the evolution process of the ablation pit in the *X*-*Z* plane; that is, the evolution process of melting and vaporization. Figure 7c–f show that as the ablation time of the laser changes, the temperature gradually decreases in the direction away from the center, and the center temperature of the laser beam is the highest. There is a small drop in the center position due to the temperature exceeding the melting point of the material, leading to the significant mass migration of the target block. The temperature field distribution shows that laser melting starts as the material melts, indicating that the amount of gasification increases with time, and the amount of melting gradually increases.

These experimental and simulation results show that the laser ablation of Al alloy targets is mainly based on the photothermal effect. Various changes will occur on the surface area of the material under different power densities when the laser is irradiated on the surface of the material. These changes include the following [31,32,33]. Melting: when a target material absorbs laser energy, its temperature rises to its melting point, causing the material to change from a solid to a liquid state. Evaporation and sublimation: if the intensity of the laser is high enough, the temperature of the material can quickly rise above the boiling point, and the material will directly change from a solid or liquid to a gas. Vaporized matter gathers near the surface of the material and ionizes to form a plasma, helping the material absorb the laser. Under the pressure of vaporization expansion, the liquid surface deforms, and pits form. When plasma with a higher degree of ionization forms, this dense plasma has a shielding effect on the laser, greatly reducing the energy density of the laser incident into the material. Solidification: after laser heating, the material’s transformation from a liquid to a solid is called solidification. After solidification, a fixed ablative morphology will be left on the target. In addition, the absorption of the laser by the target should also be considered during ablation, that is, the laser’s energy utilization rate. During laser ablation, the absorption rate of the material surface varies with the laser power density and the material surface temperature. When the material is not dissolved, the absorption rate of the laser material increases slowly with an increase in its surface temperature. When the power density is large (10^7^ W/cm^2^), the material is violently vaporized, forming a keyhole. The laser enters the keyhole for multiple reflections and absorption, dramatically increasing the absorption rate of the laser material, and the ablation depth will increase significantly [33,34].

To further demonstrate the differences in the transient force generated by single-pulse Al/Ti alloy laser ablation, the simulation results for Ti alloy laser ablation are shown in Figure S5. The temperature curve obtained from the simulation indicates that during the laser loading process, the temperature of the Ti alloy rapidly rises to approximately 2000 degrees, and then there is no significant decrease. The temperature at the ablation center of the Ti alloy target begins to drop at around 1600 degrees, indicating that the temperature has reached the melting point of the Ti alloy at this point, which enters a molten state under the action of the laser. However, the high melting point of the Ti alloy suggests that a higher laser power density is required for effective laser ablation; temperature propagation within the target is mainly related to the thermal conductivity of its material and its absorption of laser energy. The displacement change curve of the center position is similar to that of the Al alloy, but the depth of the pit at the center point is lower, indicating that under these conditions, the laser ablation power density can meet the effective ablation of both target materials, thereby generating effective pulsed force. To explore the absorption characteristics of the target materials under the laser, the reflection mode of a near-infrared spectrometer was used to test the light reflection characteristics of the two target materials in a range of 900–1700 nm (Figure S6). At a light wavelength of 1064 nm, the reflectance values of the Al/Ti alloy target materials are 45% and 4%, lower than those of high-purity alloys. This can be mainly attributed to the basic physical properties, laser energy, ultra-thin oxide layer, and roughness of the surface [35,36]. Notably, the reflectance of the Al alloy is significantly higher than that of Ti alloy; furthermore, the transient force generated during Al alloy laser ablation is also significantly higher than that of the Ti alloy. This is mainly due to the relatively high-power density of the laser ablation needed for alloy targets. At this time, the light reflection characteristics of the target material have little impact on the laser ablation effects. However, when the laser power density is low, the light reflection characteristics will greatly affect the energy utilization rate of the laser, resulting in significant differences in the laser ablation effect and significant changes in the transient force generated.

This laser ablation process demonstrates that the generation of transient force is mainly related to the formation of target physical properties, target migration mass, the environmental medium, and the laser energy utilization rate. In research on laser ablation propulsion technology, on one hand, high-efficiency light-to-force conversion is required. On the other hand, the sustainable ablation state and mass utilization rate of the target material must be considered. When the mass transfer rate of the laser ablation target material is large, the resulting transient force is also significant. However, a high mass transfer rate can decrease the laser-ablation-specific impulse. Laser propulsion performance can be measured in terms of physical parameters, such as the specific impulse, the impulse coupling coefficient, and the energy conversion factor. The specific impulse (*I*_sp_) is calculated according to the following formula:

(7) $$I_{\mathrm{sp}}=\frac{\int F_{t}dt}{mg}$$

where *F*_t_ denotes the transient force and m denotes the migrating mass of the target material.

The impulse coupling coefficient (*C*_m_) is calculated by combining the single impulse and the laser output energy (*E*); the mathematical expression is as follows:

(8) $$C_{m}=\frac{\int F_{t}dt}{E}$$

The energy conversion efficiency (*η*) can be calculated using the specific impulse and the impulse coupling coefficient; the mathematical expression is as follows:

(9) $$\eta =\frac{g}{2}C_{m}I_{\mathrm{sp}}$$

Formulas (7)–(9) show that using transient force measurements with a PVDF sensor is better for analyzing the physical processes generated by laser ablation. Nevertheless, too much laser energy and migrating mass in the target material can limit laser propulsion performance. Therefore, it is necessary to comprehensively consider both laser parameters and the physical properties of the target material to effectively enhance the performance of laser propulsion.

The experimental results for the transient force generated by Al and Ti alloy laser ablation measured with FVDF sensors demonstrate that optimizing laser parameters and target material properties can effectively improve the performance of laser propulsion. The following factors can increase the key technical parameter of specific impulse in laser propulsion. (1) Under limited conditions, we must try to increase the energy of incident lasers as much as possible. This improves laser ablation efficiency, generates high-temperature expanding gas to perform work externally, and thus produces a larger impulse. (2) We should select materials with better absorption at the laser’s corresponding wavelength. Increasing the absorption rate of laser energy means better laser energy deposition efficiency, thereby increasing the conversion efficiency of light to force and impulse generation. (3) We should select materials with a lower laser ablation energy threshold. This increases the ionization degree of the ablated target material under limited laser energy conditions, thereby generating more effective ablation effects. (4) We should select materials with more binding energy. This helps to avoid excessive migration mass during laser ablation. During laser ablation, photothermal explosions will carry many particles around the ablation area, thereby increasing unnecessary migration mass, which will reduce the specific impulse and be unfavorable for regulating and controlling thrust. (5) Considering the relationship between the laser and the ablation material comprehensively, and adjusting the appropriate laser energy and spot size to ablate the material according to its basic characteristics, can increase the specific impulse.

## 4. Conclusions

In conclusion, a novel calibration method using a PVDF piezoelectric sensor based on electromagnetic force was developed. This calibration method is simple to operate, has a wide calibration force source adjustment range, has high calibration accuracy, and is a simplified calibration device. The sensor can be used to quickly and accurately measure the transient forces generated during Al/Ti alloy laser ablation in a vacuum and air. At the same time, it can also record the transient force waveform generated during the laser ablation of a working medium, which helps to analyze the mechanisms of laser loading and laser ablation working media. This piezoelectric sensor has excellent transient force sensing performance in a vacuum and air, with a high peak voltage response, good repeatability, and a linear detection range for transient force from 0.01 N to 8.5 N. Through COMSOL Multiphysics simulation, we verified that the transient force generated during Al/Ti alloy laser ablation is mainly related to the laser power density, the laser energy utilization rate, and basic characteristics of the target material. This is the main reason for the change in transient force properties. The proposed transient force sensing strategy can be used to promote the study of laser–matter interaction mechanisms and laser propulsion technology.

## Figures and Tables

**Table 1:** Thermophysical properties of target materials.

Property (A)	6061Al Alloy	TiC4 Alloy	Parameter
Density (kg/m^3^)	2.75 × 10^3^	4.51 × 10^3^	*ρ*
Specific heat capacity (J/(kgK))	896	540	*C* *_p_*
Heat conductivity (W/(mK))	156	7.1	*k*

## Data Availability

Data are contained within the article.

## Supplementary Materials

The following supporting information can be downloaded at: https://www.mdpi.com/article/10.3390/s25092783/s1, Figure S1: Schematic diagram of calibrating a standard force source using an electronic balance; Figure S2: Voltage signal generated by laser ablation alloy target of PVDF sensor with a standard force of 0.01 N; Figure S3: Voltage signal generated by laser ablation Al alloy of PVDF sensor under different spot sizes with laser energy of 800 mJ (a), 500 mJ (b), 350 mJ (c) 150 mJ (d), and 90 mJ (e); (f) Diagram of peak voltage variation with laser energy and spot size; Figure S4: Incident light waveform of nanosecond laser; Figure S5: (a) Simulate of the temperature and center position variation relationship over time of laser ablation of Ti alloys; (b) Microscopic morphology of nanosecond laser ablation of Ti alloy under laser energy of 800 mJ and spot size of ~0.76 mm; Simulate the process of center position change in laser ablation of Ti alloy with laser energy of 800 mJ and spot size of ~0.76 mm; Figure S6: Reflectance of Ti/Al alloy in the wavelength range of 900–1700 nm.
