# Supplementary material for: Transient Force Measurement and Mechanism Analysis of Nanosecond Laser Ablation of Al/Ti Alloys Using Polyvinylidene Fluoride Sensors
Source: Sensors (Basel). 2025 Apr 28;25(9):2783. doi: 10.3390/s25092783 (PMC12074488; doi:10.3390/s25092783)
Supplement: Supplementary file 1 [file sensors-25-02783-s001.zip › sensors-3579500-supplementary.pdf]

Supplemental Document

Transient force measurement and mechanism analysis of nanosecond laser ablation of Al/Ti alloys using Polyvinylidene Fluoride sensors

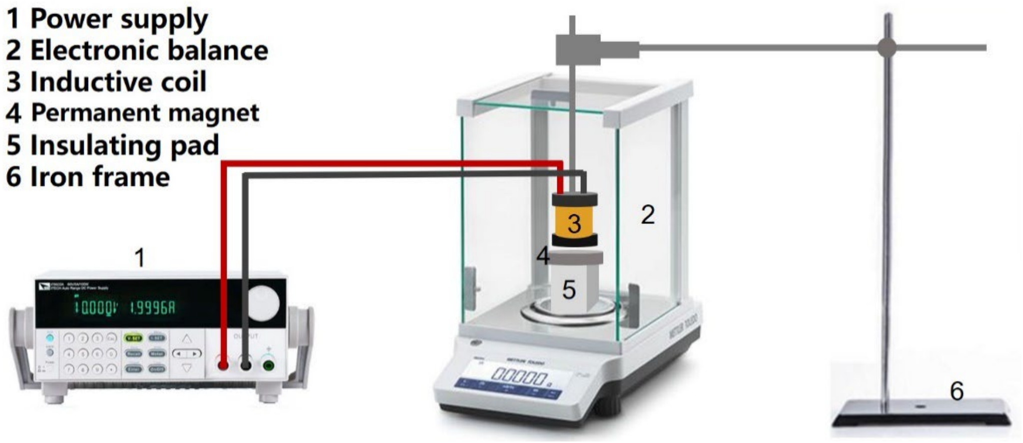

Figure S1. Schematic diagram of calibrating a standard force source using an electronic balance.

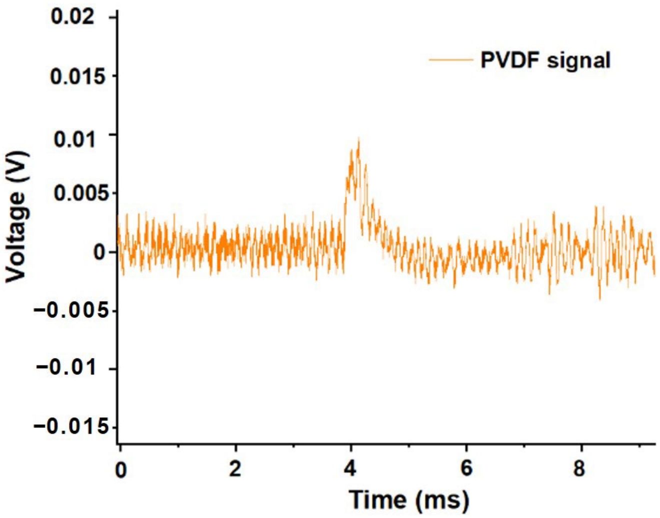

Figure S2. Voltage signal generated by laser ablation alloy target of PVDF sensor with a standard force of 0.01N.

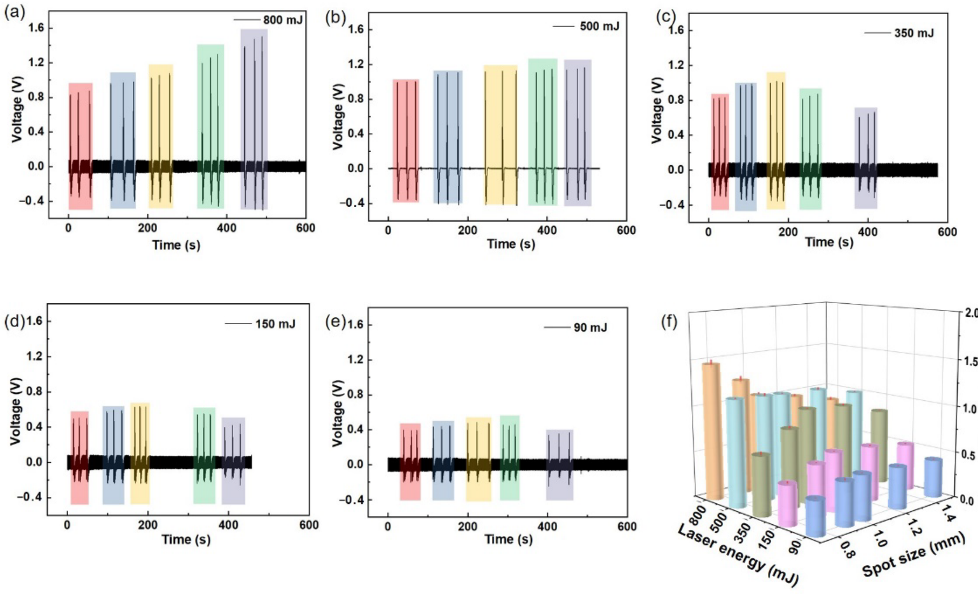

Figure S3. Voltage signal generated by laser ablation Al alloy of PVDF sensor under different spot sizes with laser energy of 800mJ(a), 500mJ(b), 350mJ(c) 150mJ(d), and 90mJ(e); (f) Diagram of peak voltage variation with laser energy and spot size.

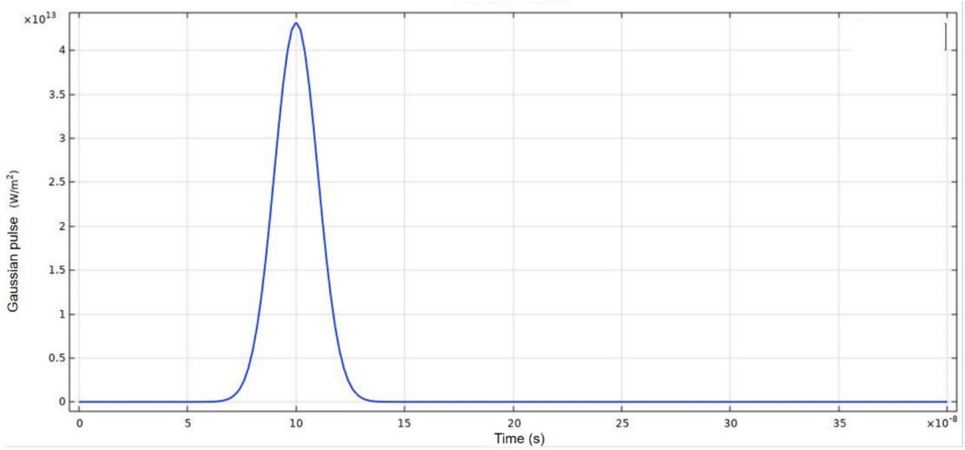

Figure S4. Incident light waveform of nanosecond laser.

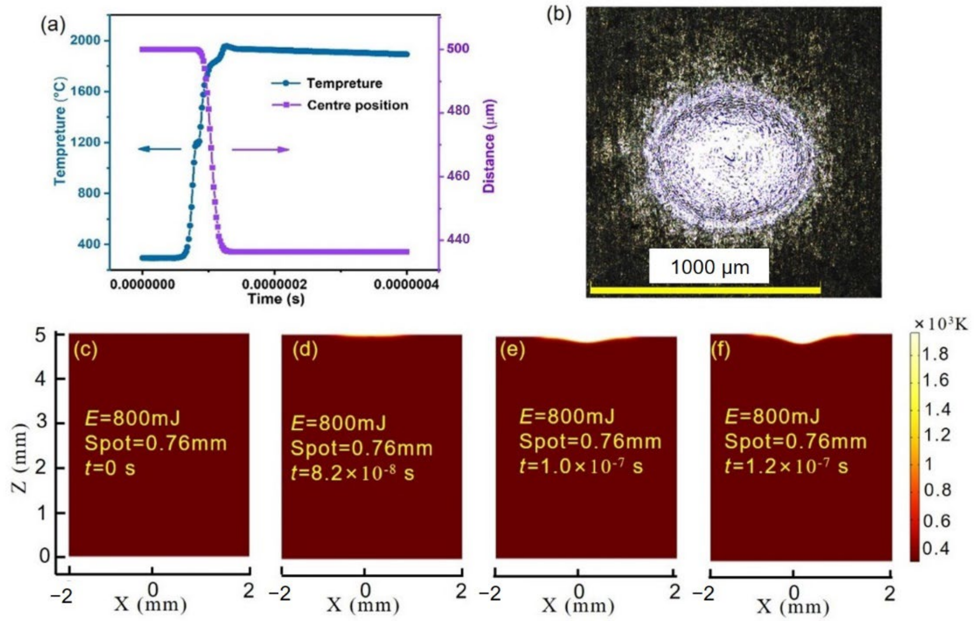

Figure S5. (a) Simulate of the temperature and center position variation relationship over time of laser ablation of Ti alloys; **(b)** Microscopic morphology of nanosecond laser ablation of Ti alloy under laser energy of 800 mJ and spot size of ~0.76 mm; Simulate the process of center position change in laser ablation of Ti alloy with laser energy of 800mJ and spot size of ~0.76mm.

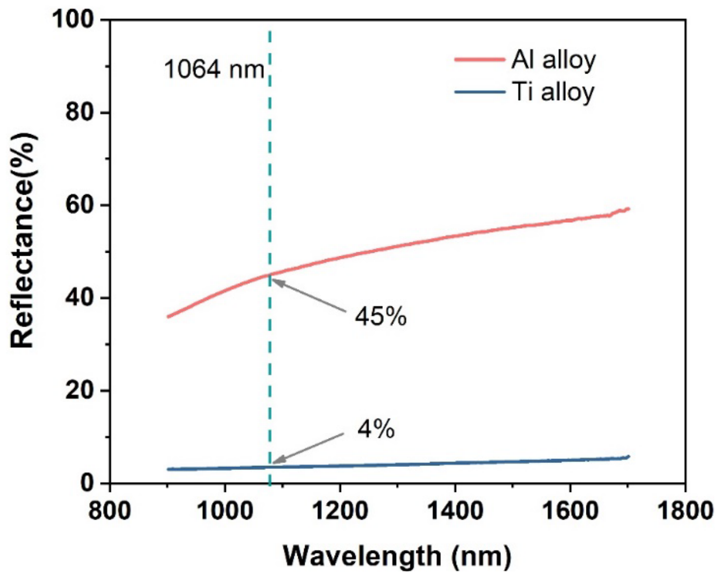

Figure S6. Reflectance of Ti/Al alloy in the wavelength range of 900-1700nm.
